# A Comprehensive Experimental Emulation for OTFS Waveform RF-Impairments

**DOI:** 10.3390/s23010038

**Published:** 2022-12-21

**Authors:** Abdelrahman Abushattal, Salah Eddine Zegrar, Ayhan Yazgan, Hüseyin Arslan

**Affiliations:** 1 Department of Electrical and Electronics Engineering, Karadeniz Teknik Üniversitesi, Trabzon 61080, Turkey; ayhanyazgan@ktu.edu.tr; 2 Department of Electrical and Electronics Engineering, Istanbul Medipol University, Istanbul 34810, Turkey; salah.zegrar@std.medipol.edu.tr (S.E.Z.); arslan@usf.edu (H.A.)

**Keywords:** OTFS, OFDM, RF-impairments, I/Q imbalances, DC-offset, phase noise, carrier frequency offset

## Abstract

The orthogonal time-frequency space (OTFS) waveform exceeds the challenges that face orthogonal frequency division multiplexing (OFDM) in a high-mobility environment with high time-frequency dispersive channels. Since radio frequency (RF) impairments have a direct impact on waveform behavior, this paper investigates the experimental implementation of RF-impairments that affect OTFS waveform performance and compares them to the OFDM waveform as a benchmark. Firstly, the doubly-dispersive channel effect is analyzed, and then an experimental framework is established for investigating the impact of RF-impairments, including non-linearity, carrier frequency offset (CFO), I/Q imbalances, DC-offset, and phase noise are considered. The experiments were conducted in a real indoor wireless environment using software-defined radio (SDR) at carrier frequencies of 2.4 GHz and 5 GHz based on the Keysight EXG X-Series devices. The comparison of the performances of OFDM and OTFS in the presence of RF-impairments reveals that OTFS significantly outperforms OFDM.

## 1. Introduction

The exponential growth in the number of connected devices created an urge to differentiate and fulfill the various requirements of the users in the network so that all are properly served properly [1]. These needs are the main driving factors behind 5G, which mainly supports three services: enhanced mobile broadband (eMBB), ultra-reliable and low latency communications (URLLC), and massive machine type communications (mMTC). These different services are achieved by using multiple OFDM numerologies [2,3,4]. The same concept is envisioned for 6G, where instead of implementing OFDM with multiple parameters, the network will be ultra-flexible and will accommodate multiple different waveforms in a single frame to meet the requirements of 6G [5].

One of the most promising waveform candidates is the OTFS waveform, which represents the information symbols in the delay-Doppler domain, where all modulated data experiences almost the same channel gain even at high mobility cases [6]. This enhances the performance of the system, which suffers from a high Doppler frequency shift compared to conventional multi-carrier techniques such as OFDM [7]. The rich scattering environment, and the mobility of the transmitter, receiver, and scatterers lead to fast variation in the time/frequency response of the wireless channel, which is very hard and expensive to estimate and compensate [8]. Additionally, the OTFS combats all of these channel effects better than most conventional schemes in high time/frequency dispersion channels. Moreover, it has been shown that OTFS achieves better BER performance compared to OFDM for mobile users with a velocity ranging between 30 and 500 Km/h [6,7].

According to the above features, OTFS has gained interest in various applications. Lately, in [9,10] OTFS is investigated for a joint radar communication (JRC) system. In [9], the authors propose an OTFS-based matched filter where it is shown that OTFS provides better tracking speed and radar distance range compared to the OFDM waveform. The authors of [11] demonstrated that the OTFS has a simply sparse structure for every possible type of prefix/suffix. In addition to this, they introduce a different structure for the OTFS, which they call the reduced-full CP (RFCP), while in [10] the authors examined OTFS performance in vehicle applications for mono-static radar. In [12,13], NOMA is integrated with OTFS to provide spectral efficiency and serve multiple users with different mobility characteristics (i.e., stationary and high mobility) in heterogeneous networks. In [14,15], the authors investigated the performance of OTFS in mm-Wave communications. It is shown that OTFS provides better robustness against high Doppler shift and phase noise that exists in mm-Wave communications. Furthermore, in [16] the author proposed multiple-mode OTFS-index modulation to enhance OTFS system BER while providing high spectral efficiency. Furthermore, the OTFS waveform is appropriate for high mobility IoT applications and wireless sensor communication networks (WSNs) due to the ability to obtain a full diversity of the channel caused by spreading the information in time and frequency block [17,18]. The 5G low-density parity-check code (LDPC) and 3GPP turbo code were utilized as channel coded-OTFS by the authors of [19]. It was shown that for high-mobility channels, 3GPP turbo code provides the best performance

However, the effect of the waveform’s physical design should be considered to have a clear understanding of the system’s performance. As a result, the impact of RF-impairments has to be defined in accordance with the results of the experimental evaluation. The research that is associated with RF-impairments is discussed in the next part.

### 1.1. Related Work

Even though the OTFS works well in demanding channel circumstances, RF-impairments must be investigated, validated, and compared to traditional waveform schemes. The PAPR of the OTFS has been analyzed in [20] and its performance has been compared to OFDM waveform. The results show that OTFS has a better PAPR performance compared to OFDM and also show that OTFS has a linear relationship with the number of Doppler bins. The degradation of OTFS BER caused by the power amplifier nonlinearity and I/Q imbalance for the 60 GHz band millimeter-wave testbed is investigated in [21]. To minimize the PAPR in a pilot embedded OTFS, a modified iterative clipping, and filtering (ICF) was proposed in [22]. Furthermore, in [23,24] the authors minimized PAPR by employing companding approaches; they showed that the μ law companding transform provides better performance than the A-law companding transform. The authors of [25] improve the PAPR by presenting an improved SeLective Mapping (SLM) method to provide secure transmission.

The I/Q imbalance impairments were discussed in [26,27]; it was demonstrated that the BER performance of the OTFS systems is worsened by I/Q imbalance impairments and has to be compensated. The authors in [28] use a deep neural network (DNN)-based technique to deal with I/Q imbalance. In [29], the authors provide a mathematical study of the I/Q imbalance as well as the direct current (DC) offset. They demonstrated that the image Doppler interference causes a reduction in the performance of the cyclic prefix OTFS (CP-OTFS) system. An innovative approach for compensating the impact of I/Q and DC offset on zero-padding OTFS was developed by the authors of [30]. Furthermore, the authors of [31] effectively compensate the I/Q imbalance for OTFS by using the index modulation (IM) approach. In [32], the authors investigated the effect that phase noise has on the performance of BER when OTFS modulation is present in 28 GHz mm-Wave communications. In addition, they demonstrated that the OTFS is more robust to phase noise compared to OFDM.

In order to implement the OTFS and study the receiver impairments’ effects, software-defined radio (SDR) is used. SDR is a radio communication system where all or most of the physical layer functions have been implemented in software. As discussed in [33], the authors implement an SDR design for the OTFS modem, and investigate the CFO and DC-offset impairments for the real indoor wireless channel. However, in this work, no mobility was considered in the experiment.

In comparison with the literature, there are only two related experimental papers. In [33], the authors only focus on the CFO and DC-offset. However, Doppler paths are randomly generated in the transmitter from a uniform distribution of random values with the specified maximum number of Doppler paths. Furthermore, the authors did not provide any results that present the effect of the DC-offset on the system’s performance. Furthermore, in [21] the authors investigate IQ imbalance and nonlinearity using a 60 GHz Testbed. The authors studied the combined effect of the impairments. Unlike [21], we provide a separate experimental investigation of the IQ imbalance and nonlinearity of the OTFS system and compare it with OFDM for the different number of delay and Doppler bins.

### 1.2. Motivation and Contribution

The waveform’s behavior is directly impacted by RF-impairments effects. Furthermore, RF-impairments including non-linearity, CFO, I/Q imbalances, DC-offset, and phase noise have been shown to significantly degrade the performance of the waveform. Since OTFS is the promising waveform for communication under high mobility conditions and exhibits resilience to narrow-band interference, the degradation due to the design of the OTFS system should be known to provide the optimal system design. Furthermore, taking into consideration one RF-impairment when designing an OTFS communication system may increase the effect of another impairment. Therefore, the OTFS system should be investigated under different RF-impairments. Thus, understanding the effects of RF impairments on system performance is one of the most significant and critical issues. Moreover, to obtain a clear view of the OTFS waveform’s performance, it is necessary to carry out an experimental study of the system.

Each work in the literature models, mathematically analyzes and/or compensates for one or two RF-impairments, which meant the result was only valid within the range of the authors’ assumptions without considering the effect of the rest of the RF impairments. Our study satisfies the need for a comprehensive experimental investigation that provides specifics on the impact that RF impairments have on the performance of the OTFS waveform. It is required to perform an experimental investigation in order to provide sufficient knowledge of the RF-impairments degradation. Furthermore, investigating each RF impairment independently helps researchers obtain a deeper comprehension of the essential compensation method to make up for these RF impairments. Therefore, the purpose of this paper is to acquire specific information on the impact that each RF impairment has on the OTFS system separately. In this study, we concentrate on uncoded OTFS for different purposes. As shown in [19], there is a trade-off between the coding gain and the diversity gain. This trade-off will affect the number of resolvable paths and could affect the evaluation results of the effect of RF impairments, especially CFO, as we will illustrate in Section 3.1. Secondly, most of the papers that discuss the related work modeled the uncoded OTFS. Thus, we follow these papers to validate our model and results.

Testing and evaluation of the RF impairments of the OTFS and OFDM waveforms are conducted in a real-time experiment using the Keysight Agilent Technologies EXA signal analyzer N9010A. The contributions of this paper are summarized as follows:

- Experimental research of the nonlinearity effect on the performance of the OTFS system has been investigated, taking into account a variety of delay bins *N* and Doppler bins *M* and comparing it with an OFDM waveform.
- The CFO influence was studied and emulated using a reverberation chamber and stirrer. The impact of CFO from LO mismatching and CFO from Doppler shift on the OTFS waveform’s performance is shown experimentally over a variety of normalized frequency offset values.
- We visualize and present the effect of the DC-offset on both the in-phase( $\gamma_{I}$ ) and quadrature ( $\gamma_{Q}$ ) components, and then we show the effect of the DC-offset on the OTFS system performance and compare it with OFDM.
- Furthermore, the phase noise of a practical 2.4 GHz CMOS voltage control oscillator (VCO) is modeled to examine its impact on the OTFS performance.
- Finally, we study the influence of the I/Q-imbalance on the performance of the OTFS while taking into account imperfections in the local oscillator. We did this by comparing the OTFS and OFDM waveforms with different values of gain imbalance ( ϵ ) and phase offset ( Δϕ ).

### 1.3. Organization

The remainder of this paper is organized as follows. Section 2 presents the modulation and demodulation of both OTFS and OFDM waveforms. Section 3 discusses the channel effects, The RF impairments’ impact, and shows the real implementation results. Finally, Section 4 concludes the work. (Notation: Matrices and column vectors are represented by bold, capital, and lowercase letters, respectively. E(.) , ${\left(.\right)}^{T}$ , ${\left(.\right)}^{H}$ , |⌊|.|⌋ and |.| represent expectation, transposition, Hermitian transposition, floor, and absolute value operations, respectively.)

## 2. Waveform Modulation and Demodulation

The waveform is known as the physical shape of information represented by a signal transmitted through the channel. The transmitted signal x(t) in a pulse-shaping system is formed by modulating data symbols $d_{n,k}$ onto time-frequency (delay-Doppler) shifted versions of a transmit pulse g(t) , i.e.,

(1) $$x\left(t\right)=\sum_{n\in Z}\sum_{k\in Z}d_{n,k}g_{n,k}\left(t\right),\;$$

with

(2) $$g_{n,k}\left(t\right)=\left(M_{kF}D_{nT}g\right)\left(t\right)=g\left(t-nT\right)e^{j2\pi kFt},\;$$

where $M_{\nu }D_{\tau }$ is the time-frequency shift operator that includes a delay (time shift) τ=nT and a modulation (frequency shift) ν=kF , *n* is the time index, *k* is the subcarrier index, *T* is the sampling period, and *F* is the sub-carrier spacing.

### 2.1. OFDM Waveform

In OFDM systems, *N* data symbols X(k),k=0,1,…,N−1 are mapped in the frequency domain, i.e., $D_{\tau }=0$ . The pulse shaping filter g(t) has a rectangular pulse shape in the transmitted lattice. Then, by using (1), the transmitted OFDM discrete time-domain signal is given by [34]

(3) $$x\left[n\right]=\frac{1}{\sqrt{N}}\sum_{k=0}^{N-1}X\left(k\right)e^{j2\pi nk/N}.\;$$

The discrete signal expression of the *n*-th received sample is given as

(4) $$y\left[n\right]=\frac{1}{\sqrt{N}}\sum_{k=0}^{N-1}H\left(k\right)X\left(k\right)e^{j2\pi n(k)/N}+w\left[n\right],\;$$

where H(k) denotes the channel coefficients, and w(n) is the zero-mean the additive white Gaussian noise (AWGN) with $\sigma^{2}$ variance $N\left(0,\sigma^{2}\right)$ .

### 2.2. OTFS Waveform

**Figure 1:**
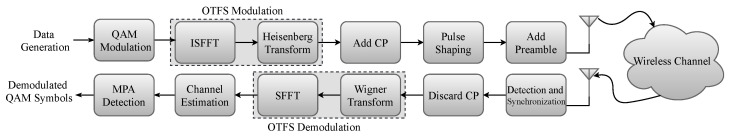
OTFS transceiver block diagram.

OTFS modulation is composed of cascaded two-dimensional (2D) transforms at both transmitter and receiver, as shown in Figure 1. At the transmitter side, the information symbols X[l,k],k=0,…,N−1,l=0,…,M−1 are mapped in the two-dimensional delay-Doppler domain from the modulation alphabet A to be transmit over NT time duration and using bandwidth B=MΔf where Δf=1/T , and *N* and *M* are the delay and Doppler bins, respectively. Then, inverse symplectic finite Fourier transform (ISFFT) using to map the N×M delay-Doppler grid points into the time-frequency plane as follows

(5) $$x\left[n,m\right]=\frac{1}{\sqrt{NM}}\sum_{k=0}^{N-1}\sum_{l=0}^{M-1}X\left[k,l\right]e^{j2\pi \left(\frac{nk}{N}-\frac{ml}{M}\right)}.$$

As illustrated by the dashed box in Figure 1, the time-frequency plane signal is transformed into a time domain signal for transmission using the Heisenberg transform, which is given by [6,7]

(6) $$x\left(t\right)=\sum_{n=0}^{N-1}\sum_{m=0}^{M-1}X\left[n,m\right]g_{tx}\left(t-nT\right)e^{j2\pi m\Delta f(t-nT)},$$

where $g_{tx}$ denotes transmit pulse shaping. Unlike OFDM where CP is added for each of *N* symbols in the frame, the CP is added for each frame in the time domain in OTFS. This considerably reduces the CP overhead. Then the signal will be transmitted through the time-varying wireless channel. The time domain received signal is expressed as

(7) $$y\left(t\right)=\int_{\nu }\int_{\tau }h\left(\tau ,\nu \right)x\left(t-\tau \right)e^{j2\pi \nu (t-\tau )}d\tau d\nu ,$$

where τ and ν denote the delay and Doppler variables, respectively. Furthermore, h(τ,ν) represent the complex channel response in delay Doppler domain. The Wigner transform is used at the receiver side to transform the time domain received signal y(t) to time-frequency domain, by matching it with the receiver pulse shaping $g_{rx}$ . Assuming that the transmit pulse shaping $g_{tx}$ and the receiver pulse shaping $g_{rx}$ satisfy the bi-orthogonality conditions then the time-frequency signal is given by [6,7,35]

(8) Y[n,m]=H[n,m]X[n,m]+W[n,m],

where W[n,m] is the noise matrix and H[n,m] is given by

(9) $$H\left[n,m\right]=\int_{\tau }\int_{\nu }h\left(\tau ,\nu \right)e^{j2\pi \nu nT}e^{-j2\pi (\nu +m\Delta f)\tau }d\nu d\tau .$$

Then, the symplectic finite Fourier transform (SFFT) is used to map the time-frequency signal in delay-Doppler domain, which is defined as follows

(10) $$\begin{array}{cc} \; & \hat{y}\left[k,l\right]=\frac{1}{\sqrt{NM}}\sum_{n=0}^{N-1}\sum_{m=0}^{N}Y\left[n,m\right]e^{-j2\pi \left(\frac{nk}{N}-\frac{ml}{M}\right)}\\ \; & =\frac{1}{\sqrt{NM}}\sum_{n=0}^{N-1}\sum_{m=0}^{M-1}x\left[n,m\right]h\left(\tau^{{}^{\prime }},\nu^{{}^{\prime }}\right)+w\left[k,l\right], \end{array}$$

Message passing (MP) detection will be used after OTFS demodulation to detect the received symbols $\hat{x}\left[k,l\right]$ from the delay Doppler received signal $\hat{y}\left[k,l\right]$ , as will be illustrated in the following subsection. This will be performed so that the symbols can be extracted from the received signal.

### 2.3. Channel Estimation and MP Detection

**Figure 2:**
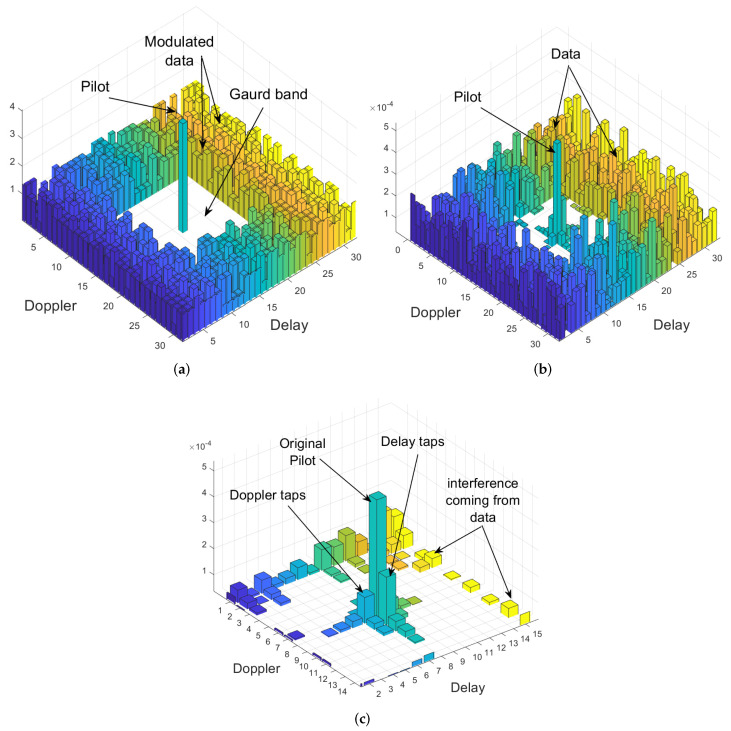
The transmitted and received signal structure in the delay-Doppler plane. (**a**) Transmitted symbols; (**b**) received signal; (**c**) received embedded pilot.

At the receiver side, the doubly-dispersive channel response has to be estimated for OTFS detection. Many methods have been proposed to estimate channel parameters, such as the flag method [36], iterative algorithm [37], and embedded pilot-aided channel estimation scheme [38]. In this paper, we adopted embedded pilot-aided channel estimation scheme, where the receiver simultaneously proceeds with a threshold approach channel estimation followed by MP data detection in the same OTFS frame. Encouraged by the embedded pilot-aided estimation, in this paper, the OTFS frame is arranged as shown in Figure 2a, where the data is distributed in the delay-Doppler domain. A guard is adopted to prevent interference between the modulated data and the embedded pilot at the receiver detection, as illustrated in Figure 2b. Finally, the delay and Doppler taps (given in Figure 2c) are estimated and passed to the MP algorithm to detect the transmitted symbols. The MP detection approach is used in this research because the representation of the communication channel in the delay-Doppler domain has a 3D (delay, Doppler, and angle) structured sparsity [39,40]. As a result, the MP algorithm provides good performance with minimal complexity for the indoor environment with a small number of channel taps [41]. After estimating the channel parameters, MP can be used to extract $\hat{x}\left[k,l\right]$ from the $\hat{y}\left[k,l\right]$ [37,42,43,44,45]. The detected received symbols are founded by evaluating the joint maximum a posteriori probability (MAP), which is represented as follows [46]:

(11) $$\hat{x}=\underset{x\in A^{NM}}{\arg\max}\mathrm{Pr}\left(x|y,H\right).$$

## 3. Channel Effects Furthermore, RF-Impairments

**Figure 3:**
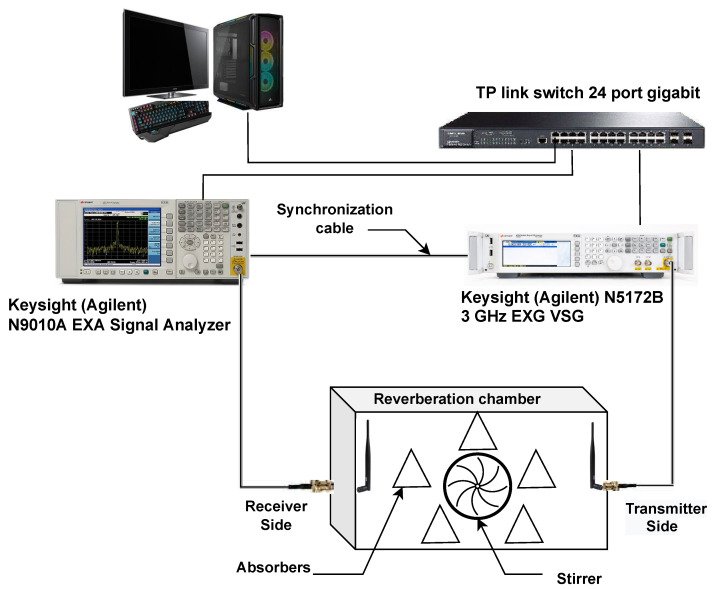
Laboratory equipment setup connection for multi-path emulation using reverberation chamber.

**Figure 4:**
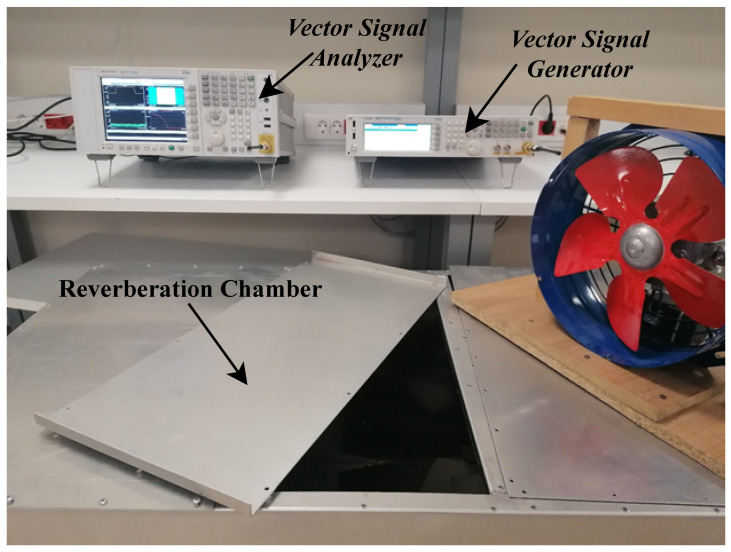
Laboratory equipment setup.

The channel effect and the various RF-impairments degrade the system’s performance. Thus, in this section, the critical RF-impairments are discussed, and their effects on OFDM and OTFS are shown and compared experimentally. In the experiment platform, the SDR device based on the Keysight EXG X-Series design is used to implement terminals. As depicted in Figure 3 and Figure 4, the N5172B VSA with 9 kHz to 1, 3, or 6 GHz frequency range with an output power +27 dBm, 900-µs switching speed. The receive antenna is connected to the N9010A EXA Keysight X-Series VSA. The setup parameters are summarized in Table 1.

### 3.1. Carrier Frequency Offset

In wireless communication, the propagating electromagnetic wave interacts with many obstacles called scatters; consequently, it is scattered, reflected, or refracted along with the propagation path. Therefore, for $L_{tap}$ different paths, $L_{tap}$ waves propagate with different delays and attenuation factors (i.e., multipath components). If the transmitter, receiver, or scatters are moving, these multipath components will be scaled in time equivalently, causing a frequency shift (narrow-band) or frequency spreading (wide-band). Thus, CFO occurs due to either Doppler effects or carrier frequency mismatching between the TX and RX oscillators [47]. Therefore, we will discuss both CFO effects in our experiment.

#### 3.1.1. CFO Due to Doppler Shift

Firstly, in our setup, to create the Doppler effect, a metallic fan (stirrer) with 40 cm × 40 cm × 20 cm dimensions and a paddle radius of 0.15 m is used to create Doppler shifts for high-speed. In order to convert these shifts into Doppler spread, a reverberation chamber with the dimensions of 120 cm × 68 cm × 55 cm is used to create more multipath and provide enough Doppler spread for the experiment. One method for generating Doppler dispersion within a reverberation chamber is to use a stirrer that moves continuously while keeping a steady receiver/transmitter setup. as shown in Figure 3 [48]. The amount of the shift in the carrier frequency $f_{D}$ is evaluated as follows

(12) $$f_{D}=\frac{V}{\lambda }\cos\theta =\frac{\omega_{p}\cdot r}{\lambda }\cos\theta ,$$

where *V*, $\omega_{p}$ , *r*, and θ represent stirring paddles’ effective linear speed, paddles angular speed, the size of the stirrer, and the effect of the angle of arrival (AOA), respectively. The λ is the wavelength of the signal in free space and is equal to $\lambda =C/f_{c}$ , where *C* is the speed of the light and $f_{c}$ is the carrier frequency. In order to have a sufficient impact on the shapes of the Doppler power spectrum, the RF absorbers are arranged in such a manner that they are positioned around the stirrer. Due to the fact that the stirrer’s blades move in opposite directions at the same effective linear speed [48].

**Figure 5:**
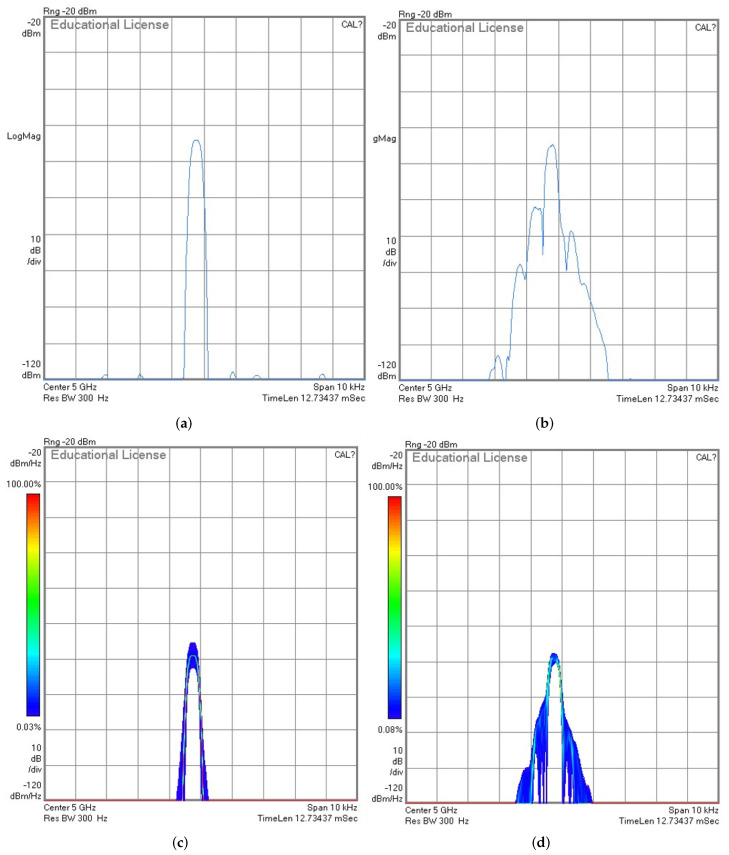

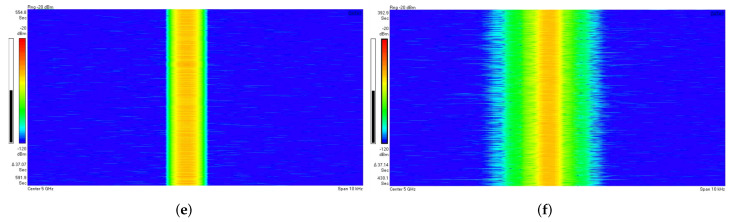
Emulation of the Doppler shift ( $f_{D}\approx 370$ Hz) effect on the 5 GHz tone using reverberation chamber. (**a**) Instantaneous spectrum stirrer-off; (**b**) instantaneous spectrum stirrer-on; (**c**) cumulative PSD when the stirrer-off; (**d**) cumulative PSD when the stirrer-on; (**e**) spectrogram when stirrer-off; (**f**) spectrogram when stirrer-on.

**Figure 6:**
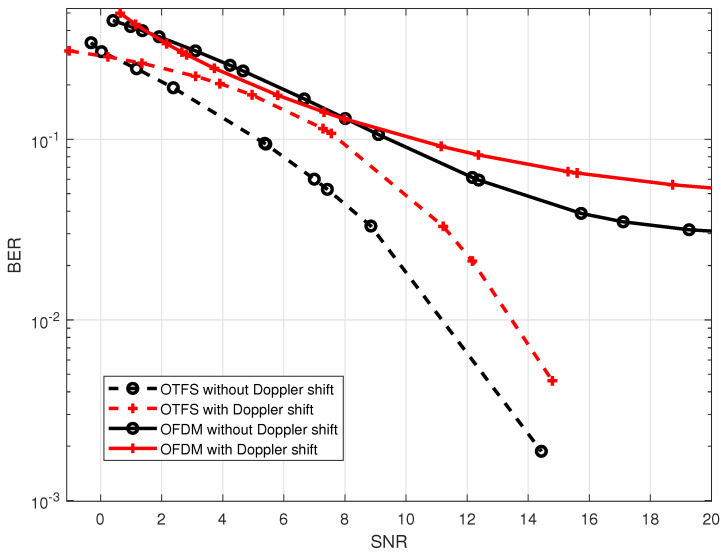
BER performance comparison between OTFS waveform with M=32,N=32 and OFDM waveform with N=1024 affected by the Doppler shift using on the central frequency is 5 GHz reverberation chamber.

Doppler shift is created through experimentation by utilizing a stirrer with dimensions of 40 cm × 40 cm × 20 cm and a paddle with a radius of r=0.15 m meters that rotates at an angler velocity of $\omega_{p}=1414.71$ rpm with AoA in the same rotation angle θ=0 , where the linear velocity is equal to V≈ 80 Km/h). According to Equation (12), this results in a Doppler shift of about $f_{D}\approx$ 370 Hz at a carrier frequency of fc=5 GHz as it shown in Figure 5. Before turning on the stirrer, it is seen that the received signal has a constant bandwidth over transmission time, however, as soon as the stirrer is turned on the spectrum enlarges taking up more bandwidth due to the spreading. When the Doppler shift is generated, the BER performance of both the OTFS and OFDM waveforms degrades, as seen in Figure 6. This degradation takes place as a result of the Doppler shift, which leads to the loss of orthogonality of the sub-carrier and eventually results in inter-carrier interference (ICI). However, as compared to the OFDM waveform, the OTFS waveform provides better BER performance. This is because the multipath components and Doppler shifts are resolvable in the delay-Doppler domain.

Assuming the resolved delays as $\tau_{i}$ and the Doppler frequency $\nu_{i}$ , the received signal is given by the following weighted summation

(13) $$y\left(t\right)=\sum_{i=1}^{L_{tap}}h_{i}x\left(t-\tau_{i}\right)e^{j2\pi \nu_{i}t}=\sum_{i=1}^{L_{tap}}h_{i}\left(M_{\nu_{i}}D_{\tau_{i}}x\right)\left(t\right),\;$$

where $h_{i}$ is the *i*-th channel complex gain and $L_{tap}$ is the total number of resolvable paths.

Note that when using the OTFS waveform, the multipath components and Doppler shifts are resolvable in the delay-Doppler domain, as seen in Figure 2c where each bin represents a tap with a specific delay and Doppler. This feature made the OTFS more suitable for rich scattering and high mobility environments than OFDM.

#### 3.1.2. CFO Due to Frequency Mismatching between the TX and RX Oscillators

Now, different frequency offsets have been implemented in the N5172B-vector signal generator (VSG) in order to replicate the carrier frequency mismatching that occurs between the TX oscillator and the RX oscillator. owing to the fact that the frequency offset of the N5172B-VSG might be adjusted.

**Figure 7:**
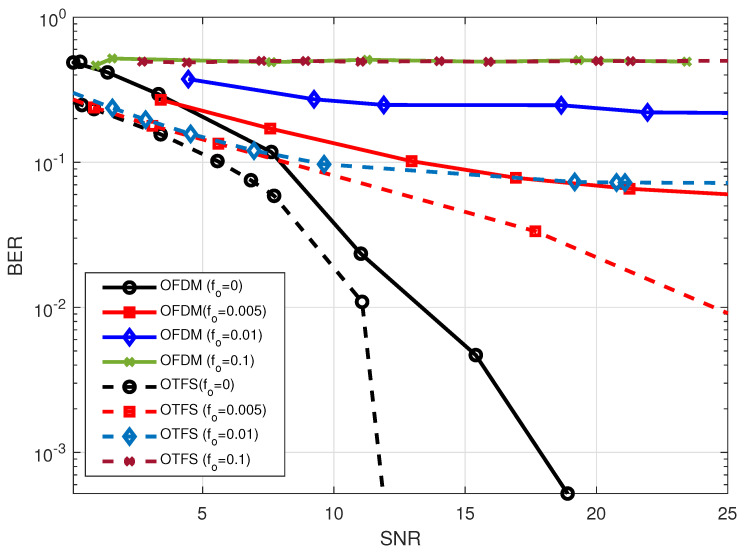
BER performance comparison between OTFS waveform with M=32,N=32 and OFDM waveform with N=1024 considering different normalized frequency offsets values in 2.4 GHz carrier without using reverberation chamber.

The effect of the Doppler shifts caused by normalized frequency offsets may be seen in the BER performance of the OFDM and OTFS, which is presented in Figure 7. As can be seen in the figure, the BER performance of both the OTFS and OFDM waveforms decays when there is an increase in the value of the mismatching frequency offset. In addition to this, it was demonstrated that OTFS has superior performance in comparison to OFDM, considering the reasons that have been discussed previously.

### 3.2. Non-Linearity Impairments

Generally, there are different nonlinearity sources in the RF front-end of communications systems, namely, high power amplifier (HPA) at the transmitter, low-noise amplifier (LNA) at the receiver, mixtures, and analog-to-digital (A/D) and digital-to-analog (D/A) converters [49]. In real-world wireless communication systems, HPAs are the most commonly used component to provide long-distance wireless transmission. According to the nonlinear input-output characteristic of HPAs, the power of the input signal should be amplified within the HPA’s linear range to prevent the HPA from being saturated and causing the out-of-band (OOB) that degrades the system performance [49]. The performance of the HPA amplifier is inversely proportional to the PAPR of the transmitted signal. As a result, the PAPR of the transmitted signal should be as minimal as possible [50]. The PAPR of the OTFS system is expressed as follows [20]

(14) $$PAPR=\frac{N\max_{k,l}{\left|x\left[k,l\right]\right|}^{2}}{E\{{\left|x\left[k,l\right]\right|}^{2}\}}.$$

Due to the fact that the PAPR is a random variable, the best way to measure and evaluate it is by using the complementary cumulative distribution function (CCDF) where the CCDF is presented as follows [20]

(15) $$\begin{array}{cc} \mathrm{Pr}\left(PAPR>\gamma_{o}\right) & =1-\mathrm{Pr}\left(PAPR\leq \gamma_{o}\right)\\ \; & \approx 1-{\left(1-e^{-\gamma_{o}}\right)}^{MN},\; \end{array}$$

where Pr(.) denotes the probability function and $\gamma_{o}$ represents the threshold level that the PAPR of the transmitted OTFS signal should not exceed.

**Figure 8:**
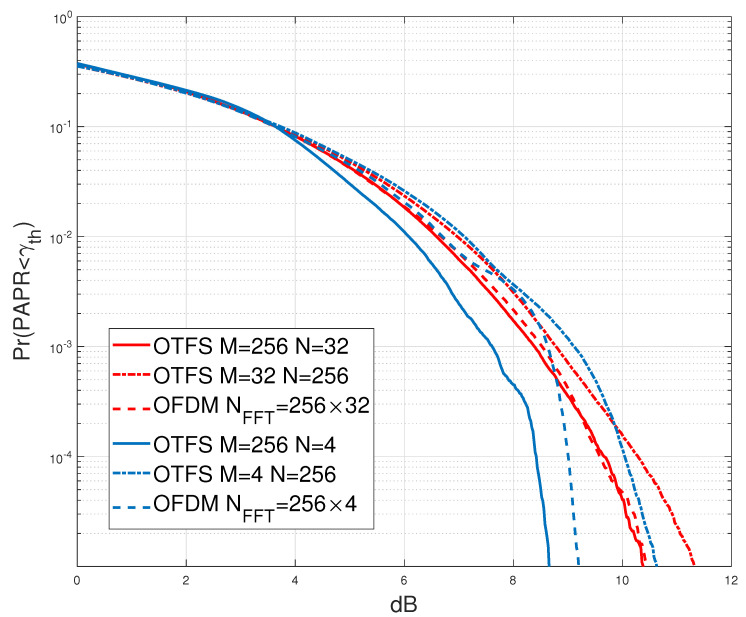
CCDF comparison of OTFS waveform and OFDM waveform at Carrier frequency 2.4 GHz.

Figure 8 compares the CCDF of the PAPR for both OTFS and OFDM with the different values of *M* and *N* subcarriers. (Note that, for comparison of *M*-subcarrier OFDM with OTFS, which have MN symbols in a frame, we consider the CCDF of the concatenation of *N* OFDM symbols.) It is shown that for the same number of sub-carrier where M>N , OTFS provides a better PAPR compared to OFDM which makes OTFS more suitable for systems that have energy consumption constraints such as IoT applications and WSNs. For example, for M=256 and N=4 , OTFS has approximately 0.5 dB less PAPR than OFDM at a probability of $10^{-5}$ . Furthermore, it is observed that as *N* increases, the PAPR of the OTFS increases, and the performance gap between the OTFS and OFDM almost vanishes. Note that in the case of N>M , it is shown that the PAPR of OTFS is the worst among all, our experimental results agree with the analytical results mentioned in [20].

### 3.3. I/Q Imbalance

In communication systems, direct-conversion receivers were used to convert RF signals to base-band signals immediately. In contrast to heterodyne receivers, direct-conversion receivers did not need the signal to be down-converted to an intermediate frequency (IF). Unfortunately, imperfections in the local oscillator (LO) might cause significant RF-impairments, such as I/Q imbalance and DC offset [51]. A local oscillator (LO) is a device that generates sinusoidal signals, which are used to represent in-phase and quadrature signals during modulation and demodulation processes. To make a cosine signal, the LO produces a sine signal and shifts it $90^{\circ }$ degrees to produce a cosine signal. Unfortunately, the signal gain created and the phase $90^{\circ }$ are not in sync in terms of their practical implementation. [47]. I/Q imbalance impairment is the combination of two effects; the first is the amplitude or gain imbalance (I) and the quadrature (Q) paths known as ϵ , and the second is the phase mismatch given by Δϕ . The following model is used to add the I/Q imbalance on the transmitted signal x(t)=I+jQ [52,53], as follows

(16) $$\begin{array}{cc} y(t) & =(1+\epsilon )\cos\Delta \phi ℜ\{x(t)\}-j(1-\epsilon )\sin\Delta \phi ℜ\{x(t)\}\\ \; & +j(1-\epsilon )\cos\Delta \phi ℑ\{x(t)\}-(1+\epsilon )\sin\Delta \phi ℑ\{x(t)\},\; \end{array}$$

where ℜ(·) and ℑ(·) symbolize the real and the imaginary part, respectively. For more simplicity, (16) can be written as

(17) $$y\left(t\right)=\alpha \cdot x\left(t\right)+\beta \cdot x{\left(t\right)}^{*},\;$$

where ${\left(\cdot \right)}^{*}$ denote complex conjugate, α=cosΔϕ+jϵsinΔϕ , and β=ϵcosΔϕ−jsinΔϕ . As it is observed in (17) that the I/Q imbalance does not exist if α=1 and β=0 .

**Figure 9:**
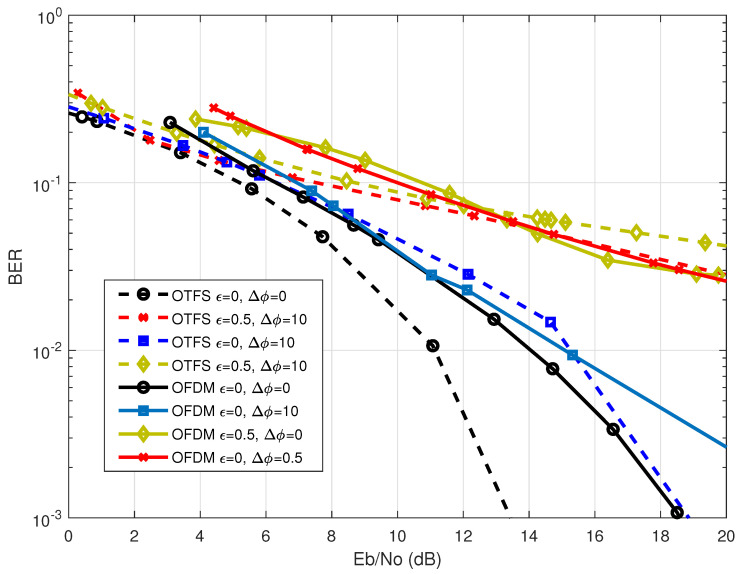
BER performance comparison between OTFS waveform with M=32,N=32 and OFDM waveform with N=1024 considering different gains imbalance ( ϵ ) and phase mismatch ( Δϕ ) I/Q imbalances at 2.4 GHz carrier frequency.

In the N5172B-VSG, there is a specification for an internal I/Q baseband generator that may be adjusted either internally or externally, depending on the application. In this experiment, we adjust the internal I/Q baseband of the N5172B-VSG to evaluate the effect of the I/Q imbalance on the performance of the OTFS system. Figure 9 shows the effect of I/Q imbalance on the average BER performance for both OTFS and OFDM systems. In general, I/Q imbalance degrades the system’s performance for both waveforms as ϵ and/or Δϕ increase, whereas the I/Q imbalances are introduced in the system, the performance directly converges to a constant error floor at certain SNR value. Beyond this value, even increasing SNR does not help in improving the BER performance as given in [26].

**Figure 10:**
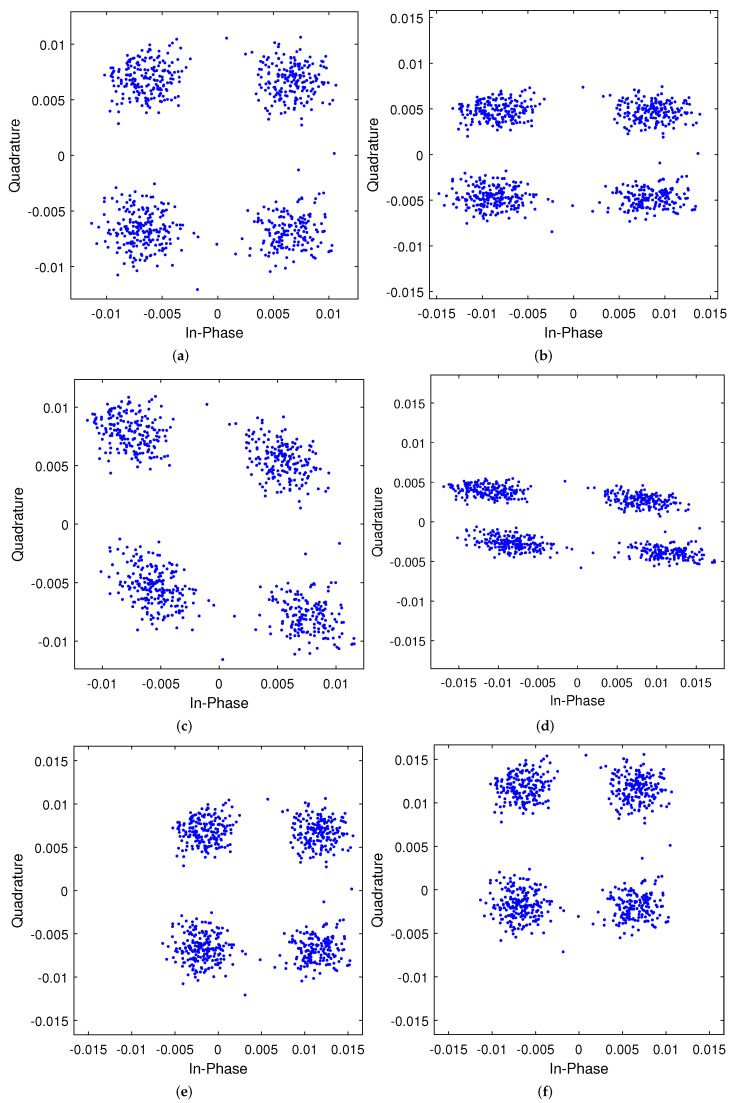
Constellation diagram shows the effect of the different I/Q and DC offset on the M=32,N=32 OTFS received information symbols of $\hat{y}\left[k,l\right]$ for 4-QAM at 2.4 GHz carrier frequency. (**a**) ϵ=0,Δϕ=0 ; (**b**) ϵ=0,Δϕ=10 ; (**c**) ϵ=0.5,Δϕ=0 ; (**d**) ϵ=0.5,Δϕ=10 ; (**e**) $\gamma_{I}=0.75,\gamma_{Q}=0$ ; (**f**) $\gamma_{I}=0,\gamma_{Q}=0.75$ .

Additionally, it can be seen that changing the value of gain ϵ causes a bad influence that is greater than that of Δϕ . This is because changing ϵ leads to narrowing the received symbols in constellations, which in turn leads to narrowing the threshold regions that the demodulation should use to distinguish the received symbols. In other words, changing ϵ causes a more negative effect than changing Δϕ , Since the points in the constellation are now quite close to each other, while changing Δϕ causes symbols to shift without changing the distance between neighboring points as shown in Figure 10b–d.

### 3.4. DC Offset

The DC offset is also caused by the imperfection of the LO in direct-conversion receivers. where it is induced due to the leakage self-mixing of LO and transistor mismatch in the RF components [29]. DC offset will result in a shift on the symbols used in the constellation diagram (I/Q plane), and as shown in Figure 10d,e, this shift might occur on the I-component ( $\gamma_{I}\sqrt{E_{s}}$ ), the Q-component $\gamma_{Q}\sqrt{E_{s}}$ , or both of them [51]. Where $E_{s}$ is energy per symbol.

**Figure 11:**
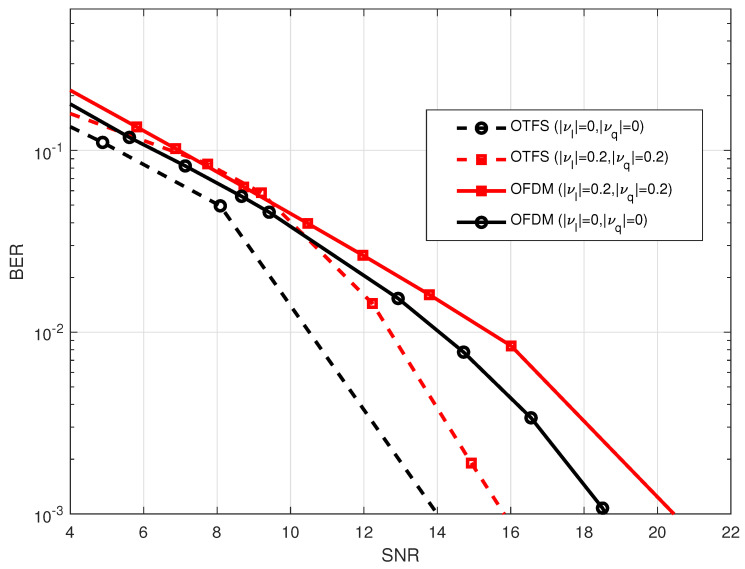
Comparison of the BER performance between OTFS waveform with M=32,N=32 and OFDM waveform with N=1024 considering different DC-offest in both I-component ( $\gamma_{I}$ ) and Q-component ( $\gamma_{Q}$ ) at 2.4 GHz carrier frequency.

The comparison of the impact of the DC-offset on the BER performance of OTFS and OFDM is shown in Figure 11. In general, the BER performance of the OTFS waveform is superior to the OFDM waveform under the effect of the DC offset. Furthermore, it demonstrates that the BER performance degrades as the value of the DC-offset increases, and this will happen with nearly the same amount of effect for both OTFS and OFDM waveforms. Specifically, if the amount of the DC-offset is raised by around $20\%\sqrt{E_{s}}$ , then the system will need approximately SNR≈2 dB more in order to maintain the same BER $=10^{-3}$ . As demonstrated, the effect of the DC-offset has a smaller impact on the system when the SNR is low, but it becomes more noticeable as the SNR rises. This is because the DC-offset expresses in the form of interference in the center of the transmission frequency spectrum, and as the SNR rises, the interference’s impact on the system’s overall performance accumulates.

**Figure 12:**
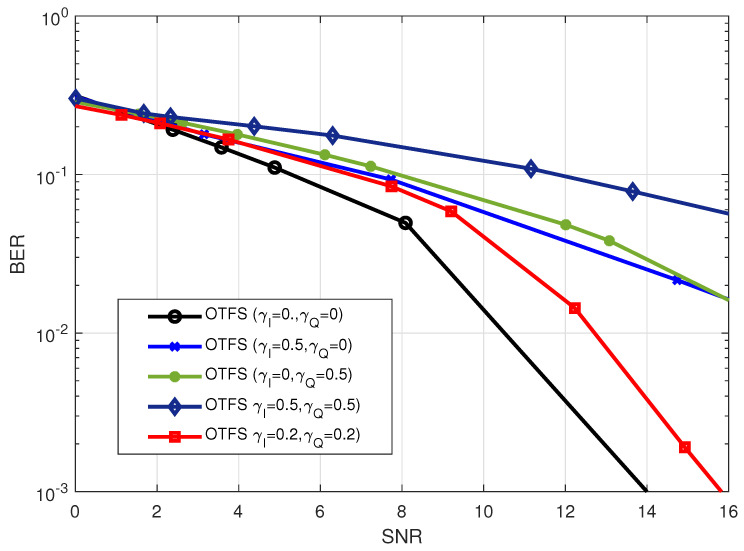
BER performance for OTFS waveform with M=32,N=32 under different values of DC-offset in both I-component ( $\gamma_{I}$ ) and Q-component ( $\gamma_{Q}$ ) at 2.4 GHz carrier frequency.

The impact that varying values of the DC-offset have on the performance of the OTFS waveform is seen in Figure 12. According to the results, the degradation in system performance may be attributed to an increase in the DC-offset values. Additionally, it was shown that whether the in-phase DC-offset or the quadrature DC offset was used, the system had the same influence on the performance of the system.

### 3.5. Phase Noise

**Figure 13:**
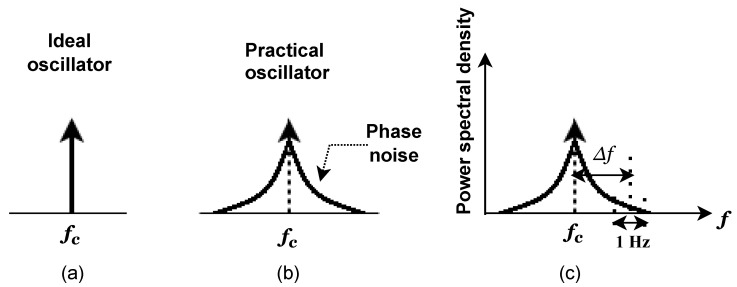
Phase noise of the local oscillator. (**a**) Ideal oscillator, (**b**) practical oscillator, and (**c**) phase noise power level in dBc/Hz with Δf offset.

When a local oscillator in a transceiver is unable to create pure sinusoidal waves in conformance with the Dirac spectrum, phase noise is produced, as shown in Figure 13a,b. The frequency spectrum and timing properties of the oscillator output induce large adjustments as a direct result of PN’s influence [47].

In most cases, designers typically define PN in the frequency domain, using a bandwidth of one Hz and an offset of one Δf from the carrier [47]. The power of the PN signal throughout this bandwidth is normalized in relation to the power of the carrier in dBc/Hz units, as illustrated in Figure 13c.

**Figure 14:**
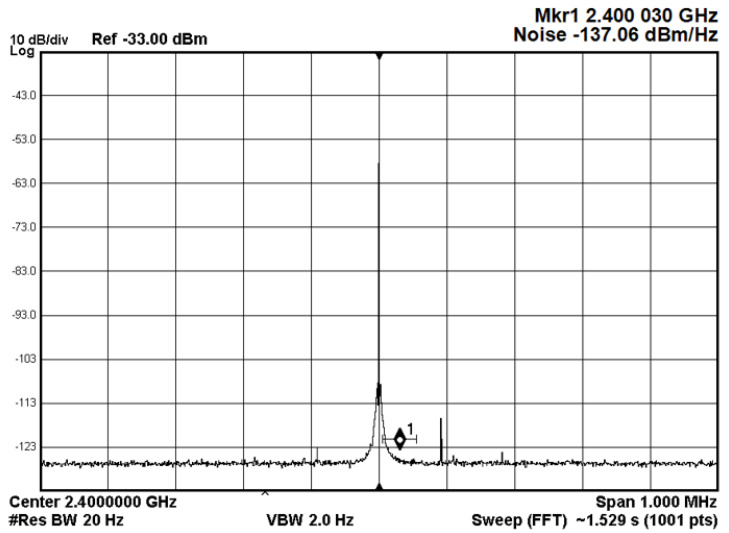
The phase noise of the VSA local oscillator.

Both the N5172B VSG and the N9010A EXA Keysight X-Series VSA had amazingly low phase noise in our experiment. The phase noise of the VSA’s local oscillator is shown in Figure 14. According to what has been seen, the phase noise of the local oscillator is equivalent to −137.06 dBm/Hz at a 30 Hz frequency offset.

**Figure 15:**
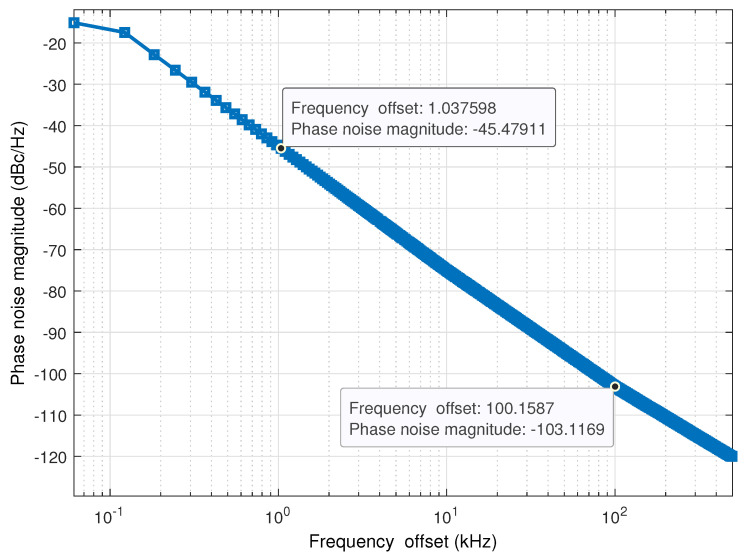
Phase noise model for 2.4 GHz CMOS VCO.

**Figure 16:**
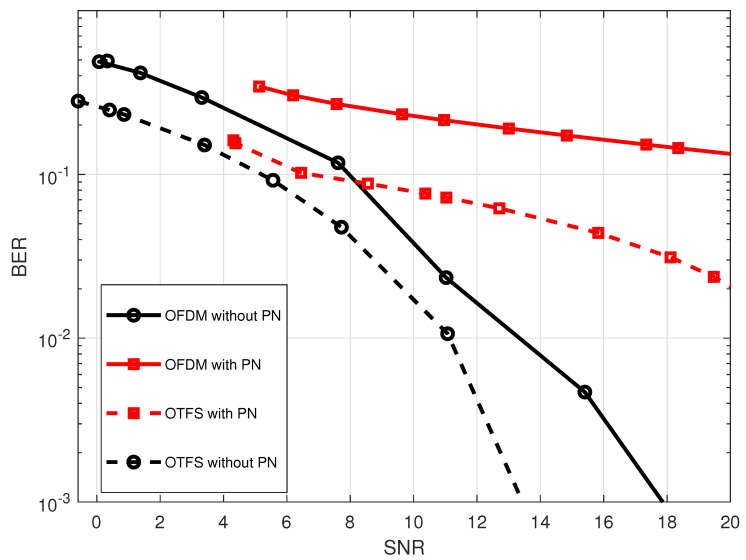
Comparison of the effect of the phase noise on the BER performance for both OTFS waveform with M=32,N=32 and OFDM waveform with N=1024 at 2.4 GHz carrier frequency.

The influence of phase noise will not be seen on the performance of the waveform systems because the level of phase noise is very low. Therefore, we have introduced phase noise at the receiver, as illustrated in Figure 15. Furthermore, this modeling of the phase noise already exists on the 2.4 GHz complementary metal-oxide-semiconductor voltage control oscillator (2.4 GHz CMOS VCO) [54]. The phase noise impact on OTFS and OFDM BER performance is shown in Figure 16 Phase noise has been proven to have a detrimental effect on both the system’s performance and the orthogonality of the subcarriers, resulting in ICI. In contrast to OFDM, the OTFS waveform is more resistant to the phase noise effect. The observed results are consistent with the conclusions presented in [32].

## 4. Conclusions

This paper emulates and compares the RF-impairments of the OTFS waveform to OFDM impairments using SDR. The experiments were conducted in a real indoor wireless environment, where the metallic structure of the building introduced enough multipath, the Doppler shift was induced by the stirrer, and the impairments were either inherently produced inside the devices or added before transmission. The BER performance of the OTFS modulation was superior to that of the OFDM under doubly dispersive channel. The CCDF of the PAPR of OTFS is shown to vary with the lattice structure in the delay-Doppler domain, and under the M>N condition, OTFS provides a better PAPR compared to OFDM. In addition, the I/Q-imbalance and DC-Offest impairments were explored, and the results showed that OTFS and OFDM are impacted in a manner that is approximately identical to one another. In addition, phase noise mitigation and OTFS give a higher level of phase noise resistance in comparison to OFDM. These findings provide an understanding of how and when to choose the waveform that is best appropriate for the characteristics of a particular channel.

In future work, the practical pulse shaping strategies should be taken into consideration, and methods should be provided to minimize the impacts of each RF impairment on the OTFS waveform. Furthermore, channel estimation and coding overhead, complexity, and performance gain are critical issues that need to be taken into consideration with a detailed analysis for different waveforms.

## Figures and Tables

**Table 1:** Setup parameters.

Symbol	Parameters	Value (OTFS)	Value (OFDM)
$f_{c}$	Carrier frequency	2.4 GHz, 5 GHz	2.4 GHz, 5 GHz
*M*	Number of subcarriers	432, 256	256, 1024
*N*	Number of symbols	432, 256	1
$T_{s}$	Symbol duration	10μ s	
$M_{mod}$	Modulation order	4-QAM	
$\Delta f_{s}$	Sub-carrier spacing	100 KHz	
$f_{o}$	Normalized frequency offset	0,0.05,0.1,0.3	
ϵ	I/Q gain imbalance	0%,50%	
Δϕ	I/Q phase imbalance	$0^{\circ },\;10^{\circ }$ degree	
$\gamma_{I},\gamma_{Q}$	DC-offset	( $0\%,\;20\%,\;50\%,\;75\%)\sqrt{E_{s}}$	

## Data Availability

Not applicable.

## Abbreviations

The following abbreviations are used in this manuscript:

5G	Fifth Generation
6G	Sixth Generation
A/D	Analog-to-Digital
BER	Bit Error Rate
CCDF	Cumulative Distribution Function
CFO	Carrier Frequency Offset
CP	Cyclic Prefix
D/A	Digital-To-Analog()
DC	Direct Current
eMBB	Enhanced-Mobile Broadband
I/Q imbalance	In-Phase And Quadrature Imbalance
ICF	Iterative Clipping And Filtering
ICI	Inter-Carrier Interference
ISFFT	inverse symplectic finite Fourier transform
ISI	Inter-Symbol Interference
JRC	Joint Radar Communication
LNA	Low-Noise Amplifier
LS	Least Squares
MIMO	Multi-Input Multi Output
mMTC	Massive Machine Type Communications
mm-Wave	millimeter wave
MP	Message Passing
NOMA	Non-Orthogonal Multiple Access
OFDM	Orthogonal Frequency Division Multiplexing
OTFS	Orthogonal Time-Frequency Space
PA	Power Amplifier
PAPR	Peak-To-Average Power Ratio
PSW	Prolate Spheroida Waveform
QAM	Quadrature Amplitude Modulation
RF	Radio-Frequency
SDR	Software-Defined Radio
SFFT	Symplectic Finite Fourier Transform
SLM	improved SeLective Mapping
URLLC	Ultra-Reliable And Low Latency Communications
VSA	Vector Signal Analyzer
VSG	Vector Signal Generator
RCF	Raised-cosine filter
LO	Local oscillator
HPA	high power amplifier
OOB	out-of-band
AWGN	additive white Gaussian noise
CMOS	complementary metal-oxide-semiconductor
VCO	voltage control oscillator
